# In vitro study on the effects of exogenic fibrolytic enzymes produced from *Trichoderma longibrachiatum* on ruminal degradation of olive mill waste

**DOI:** 10.5194/aab-65-79-2022

**Published:** 2022-02-22

**Authors:** Khalil Abid, Jihene Jabri, Hela Yaich, Atef Malek, Jamel Rekhis, Mohamed Kamoun

**Affiliations:** Animal Nutrition Laboratory, National School of Veterinary Medicine Sidi Thabet, University of Manouba, Manouba, Tunisia

## Abstract

Olive mill waste is low-quality feed and rarely used in ruminant nutrition because of its high lignocellulose content, the existence of anti-nutritional factors such as total polyphenol and condensed tannin, and low protein contents. This in vitro research was conducted to valorize this waste (crude olive cake, extracted olive cake, and olive leaves) using an exogenous fibrolytic enzyme produced from *Trichoderma longibrachiatum* in ruminal nutrition. The enzymatic activity of this additive was 1161 units of endoglucanase per millilitre, 113 units of exoglucanase per millilitre, and 2267 units of xylanases per millilitre. This treatment was applied by spraying substrates with four doses: 0 (control), 1 (low), 2 (medium), and 4 $µL\;g^{-1}$ $µL\;g^{-1}$ (high) of dry matter olive mill waste in an air-conditioned room at 26 ${}^{\circ }C$ for 12 h before in vitro incubation.

For the crude olive cake, this additive at high doses increased degradation of 14 % of cellulose and 8 % of hemicellulose compared with the control at 12 h before the in vitro incubation. Consequently, it increased dry matter solubility and reduced sugars at this period compared to the control. Upon ruminal incubation, the high dose of exogenous fibrolytic enzyme increased the gas production from the immediately soluble fraction and insoluble fraction, the rate of gas production for the insoluble fraction, the dry matter degradability by 26 %, the organic matter degradability by 24 %, the metabolizable energy value by 28 %, and the microbial crude protein production by 24 % compared with the control. For olive leaves, an exogenous fibrolytic enzyme at medium dosage can also hydrolyse the hemicellulose compound, release fewer sugars, and increase dry matter solubility compared with the control at 12 h before the in vitro incubation.

Upon in vitro incubation, the medium dose increased the gas production from immediately soluble and insoluble fractions, the rate of gas production for the insoluble fraction, the dry matter degradability by 13 %, the organic matter degradability by 11 %, the metabolizable energy value by 12 %, and the microbial crude protein production by 12 % compared with the control. However, the highest dose altered the gas production from insoluble fractions and decreased microbial crude protein production by 6 % compared with the control. Under the same conditions, an exogenous fibrolytic enzyme applied to extracted olive cake did not produce any effect in the chemical composition and nutritional value. These results showed clearly that effectiveness of exogenous fibrolytic enzyme varied with incubated waste. Increasing the nutritional value of crude olive cake and olive leaves using an exogenous fibrolytic enzyme can encourage breeders to use this waste as feed at a low cost in animal nutrition. This valorization of waste is a good solution to reduce pollution of soils and groundwater caused by throwing out this polluted waste into the environment.

## 1 Introduction

The Mediterranean countries contribute 92 % of the world's production of olive oil (Tufariello et al., 2019). The olive oil extraction generates a gigantic quantity of solid waste like crude olive cake and olive leaves (Molina-Alcaide and Yáñez-Ruiz, 2008; Tufariello et al., 2019). The majority of this waste is thrown out in the nearby environment. This waste decomposes over a long period of time (years) and still presents economical (i.e. the cost of getting rid of the waste) and environmental problems in soil and groundwater (Molina-Alcaide and Yáñez-Ruiz, 2008; Dermeche et al., 2013; Fadel and El-Ghonemy, 2015; Berbel and Posadillo, 2018; Chebaibi et al., 2019). The fresh or well preserved waste of olive oil production can be utilized in ruminant feeding with no adverse effects on animal health (Awawdeh and Obeidat, 2013; Fadel and El-Ghonemy, 2015; Mannelli et al., 2018; Abid et al., 2020). However, this waste when left in the open quickly becomes rancid and cannot be eaten by animals (Molina-Alcaide and Yáñez-Ruiz, 2008). This waste is characterized by high fibre contents and the existence of anti-nutritional factors, particularly total polyphenol and condensed tannin (Molina-Alcaide and Yáñez-Ruiz, 2008). The effect of tannins varies according to their concentration, ranging from beneficial effects to toxicity and death in high doses (Makkar, 2003). The fibrous feed can be digested by the rumen microorganisms, but in most cases only 10 %–35 % of the gross energy ingested is available as net energy (Varga and Kolver, 1997).

Olive cake is the major solid waste product from the olive oil extraction process. It represent about 30 % to 40 % of the weight of processed biomass in the olive mill (Molina-Alcaide and Yáñez-Ruiz, 2008). This solid waste is a mixture of the olive skin, the crushed pulp residues, and the small fragments of the olive kernel shell (Berbel and Posadillo, 2018). The use of olive cake as ruminant feed can reduce the pollution caused by this waste and improve the quality of ovine and bovine meat and milk in particular. It increases unsaturated fatty acids, which have positive effects on human health (Hamdi et al., 2016; Kotsampasi et al., 2017; Mannelli et al., 2018; Symeou et al., 2019; Chiofalo et al., 2020; Neofytou et al., 2020). Olive leaves are the solid waste product of separated and clean olives before oil extraction. It represents around 5 % of the weight of the biomass processed in the olive mill (Hukerdi et al., 2020). The use of olive leaves as ruminant feed especially improves the quality of goat and ovine meat and milk. It improves the unsaturated fatty acid profile (Abbeddou et al., 2011; Hukerdi et al., 2020). To improve the nutritional value of this solid waste several approaches have been used, such as chemical treatments (Fegeros et al., 1995; Molina-Alcaide and Yáñez-Ruiz, 2008; Awawdeh and Obeidat, 2013) and biological treatments with white-rot fungi (Neifer et al., 2013), *Aspergilllus, Trichoderma* and* Saccharomyces* (Fadel and El-Ghonemy, 2015). The biological treatments are more effective than the chemical treatments due to their greater substrate specificity and their lower pollution effects (Misra et al., 2007). The treatment of fibrous feed by biological additives like exogenic fibrolytic enzymes (EFE) improved the nutritional value, growth performance, milk production, and economic returns of ruminants (Mohamed et al., 2013; Abid et al., 2019b, 2020). Therefore, the objective of this research is to valorize olive mill waste by supplementing ruminant nutrition with EFE.

## 2 Materials and methods

### 2.1 Sampling and chemical analysis

Crude olive cake, extracted olive cake, and olive leaves samples were collected from eight olive mills in Sfax (southern Tunisia). Three samples of each by-product in each oil mill are used. These mills used three phase centrifugation processes (Tamasi et al., 2016). The dry matter content of the samples was produced by oven-drying until it reached a constant weight (AOAC 930.15). Then samples were ground through a mill using a 1 mm sieve and a Cyclotec 1093 Sample Mill (Tecator, Höganäs, Sweden). The crude protein (CP) (AOAC 954.01), ether extract (EE) (AOAC 920.39), and ash (AOAC 942.05) of samples were determined following the method defined by AOAC (1995). Neutral detergent fibre (NDF), acid detergent fibre (ADF), and acid detergent lignin (ADL) were determined by using a fibre analyser (ANKOM220, ANKOM Technology, Macedon, New York, USA) following the method defined by Van Soest et al. (1991). Hemicellulose and cellulose were calculated by the difference between NDF and ADF and between ADF and ADL respectively (Van Soest et al., 1991).

The nitrogen-free extract was calculated by using the formula of NRC (2001) according to Eq. (1):

1 $$\begin{array}{rl} & \text{Nitrogen free extract (\%)}=\\ & 100-(\text{EE}\;(\%)+\text{CP}\;(\%)+\text{NDF}\;(\%)+\text{Ash}\;(\%)). \end{array}$$

Total phenols, total tannins, condensed tannins, and hydrolysable tannins compounds were determined following the protocol of Makkar et al. (1993, 2000). Total phenols and total tannins were determined by using a Folin–Ciocalteu substrate with spectrophotometry at 725 nm of absorbance. Condensed tannins were determined by using the acid–butanol–HCl–Fe method with spectrophotometry at 550 nm of absorbance. Hydrolysable tannins were calculated by the difference between total tannins and condensed tannins. Reducing sugars was realized following the protocol of Miller (1959). The soluble dry matter was realized following the protocol of Elwakeel et al. (2007). Briefly, samples of 1 g dry matter of each substrate were poured into glass flasks. Then 100 mL of distilled water was added. This mixture was stored for 12 h in an air-conditioned room at 26 ${}^{\circ }C$ . After incubation, residues were recovered by filtration with Whatman 541 filter paper (Whatman Scientific Ltd, Maidstone, Kent, England). DM losses were determined according to the methods described by AOAC (1995).

### 2.2 Treatment of olive mill waste using an exogenous fibrolytic enzyme

The exogenous fibrolytic enzyme used in this study was a liquid mixture of two fibrolytic enzymes, cellulase plus and xylanase plus (Dyadic International Inc., Jupiter, Florida, USA), used in equal volume. Both enzymes were obtained by the fermentation of non-recombinant* Trichoderma longibrachiatum*. The mixture was diluted in distilled water at three different concentrations, either 1 µL mixture preparation per 0.2 mL, 2 µL mixture preparation per 0.2 mL, or 4 µL mixture preparation per 0.2 mL. The mixture treatment was applied by spraying one of the enzyme solutions (or only distilled water in the control) on the olive mill waste at 0.2 mL of solution per gram of dry matter olive mill waste in an air-conditioned room at 26 ${}^{\circ }C$ for 12 h before in vitro incubations. Thus, the final concentration of this additive was 0 (control), 1 (low), 2 (medium), and 4 $µL\;g^{-1}$ (high)  dry matter olive mill waste.

The enzymatic activity of the mixture before dilution in distilled water and before treatment of substrates was analysed in triplicate at a pH of 6.6 and a temperature of 39 ${}^{\circ }C$ , to simulate a normal rumen environment. The endoglucanase activity and exoglucanase activity were measured according to the methods of Wood and Bhat (1988) by using carboxymethyl cellulose sodium salt and cellulose as substrates (Sigma Chemical Co., Saint-Louis, MO, USA) respectively. The xylanase activity was determined according to the methods of Biely and Poutanen (1992) by using oat spelt xylan as substrate (Sigma Chemical Co., Saint-Louis, MO, USA). The enzymatic activity of the mixture before dilution in distilled water and treatment of substrates used in this study had 1161 units of endoglucanase per millilitre, 113 units of exoglucanase per millilitre, and 2267 units of xylanases per millilitre.

### 2.3 Pre-incubation effects

After 12 h of treatment with EFE, solubilize dry matter, crude protein, cellulose, hemicellulose, lignin, total phenols, and reduced sugars of treated substrate were analysed in triplicate.

**Table 1:** Chemical composition ( $g\;{\mathrm{kg}}^{-1}$ dry matter).

	Crude olive cake			Extracted olive cake			Olive leaves		
	Mean	Min	Max	Mean	Min	Max	Mean	Min	Max
Dry matter ( $g\;{\mathrm{kg}}^{-1}$ fresh matter)	475	436	501	903	892	915	491	474	510
Crude protein	89	85	94	96	92	103	102	97	106
Ether extract	67	62	70	2	1	3	19	17	23
Neutral detergent fibre	579	562	591	700	687	722	402	388	410
Acid detergent fibre	461	445	478	599	589	609	251	241	259
Acid detergent lignin	222	214	230	320	314	329	104	100	110
Hemicellulose	118	102	130	101	92	115	151	141	160
Cellulose	239	226	255	279	267	289	147	138	156
Ash	116	102	122	157	147	165	74	70	78
Nitrogen-free extract	149	136	161	45	35	55	403	397	413
Reduced sugar	21	18	24	9	7	11	57	51	62
Total phenols	7	5	8	6	4	7	77	71	83
Total tannin	5	4	6	4	3	5	66	63	70
Condensed tannin	3	2	4	2	1	3	58	53	63
Hydrolysable tannins	2	1	3	2	1	3	8	6	10
Dry matter solubility	31	28	33	15	13	18	65	60	71

Min is the minima of the analysed individual fractions in the by-product, Max is the maxima of the analysed individual fraction in the by-product.

**Table 2:** Influence of exogenous fibrolytic enzymes on chemical composition of olive mill waste after pre-incubation.

Item	0 µL	1 µL	2 µL	4 µL	SEM	p value	Linear	Quadratic
Crude olive cake								
Cellulose	118 ${}^{a}$	101 ${}^{b}$	102 ${}^{b}$	100 ${}^{b}$	2.2	0.04	0.04	0.49
Hemicellulose	239 ${}^{b}$	235 ${}^{b}$	226 ${}^{\text{ab}}$	221 ${}^{b}$	4.0	0.02	0.01	0.51
Lignin	222	224	220	224	3.3	0.80	0.69	0.76
Crude protein	89	90	89	91	2.1	0.87	0.79	0.88
Total phenols	7.3	7.1	7.2	7.1	0.5	089	0.93	0.87
Reduced sugar	21 ${}^{b}$	26 ${}^{\text{ab}}$	29 ${}^{a}$	31 ${}^{a}$	1.3	0.04	0.03	0.61
Dry matter solubility	31 ${}^{b}$	33 ${}^{b}$	37 ${}^{\text{ab}}$	40 ${}^{a}$	2.2	0.04	0.02	0.43
Extracted olive cake								
Cellulose	101	100	99	104	2.1	0.89	0.91	0.88
Hemicellulose	279	272	275	273	4.1	079	0.88	0.79
Lignin	320	318	321	317	3.9	0.91	0.86	0.79
Crude protein	96	97	95	95	3.5	0.87	0.79	0.77
Total phenols	6.0	6.2	6.9	6.0	0.4	0.77	0.81	0.78
Reduced sugar	9	10	9	10	0.4	0.69	0.87	0.60
Dry matter solubility	15	16	16	15	1.1	0.87	0.79	0.88
Olive leaves								
Cellulose	151	149	145	142	2.3	0.07	0.06	0.56
Hemicellulose	147 ${}^{a}$	145 ${}^{a}$	131 ${}^{b}$	127 ${}^{b}$	1.0	0.03	0.02	0.51
Lignin	104	103	101	104	2.2	0.87	0.91	0.89
Crude protein	102	100	105	97	3.9	0.59	0.65	0.51
Total phenols	77.0	75.6	76.0	57.8	1.1	0.63	0.79	0.45
Reduced sugar	57 ${}^{b}$	61 ${}^{b}$	71 ${}^{a}$	75 ${}^{a}$	0.9	0.04	0.03	0.44
Dry matter solubility	65 ${}^{b}$	67 ${}^{b}$	82 ${}^{a}$	85 ${}^{a}$	3.1	0.04	0.02	0.52
Item	Dose		Substrate		Dose × substrate			
p value								
Cellulose	0.02		<0.001		0.01			
Hemicellulose	0.04		<0.001		0.01			
Lignin	0.57		<0.001		0.78			
Total phenols	0.67		<0.001		0.79			
Reduced sugar	0.04		<0.001		0.02			
Dry matter solubility	0.04		<0.001		0.03			

${}^{\text{a, b, c}}$ Different letters in the same row indicate that the difference is significant ( p<0.05 ). SEM is the standard error of the mean.

### 2.4 Incubation effects

Two rumen-cannulated Holstein cows ( 650±20 kg body weight) were used to collect rumen fluid before the morning meal via an electric pump. These cows received two meals composed by 8 $\mathrm{kg}\;d^{-1}$ of oat hay and 2 $\mathrm{kg}\;d^{-1}$ of a commercially available concentrate. The rumen content of these two cows was mixed and filtered through four layers of cheesecloth under a continuous flow of ${\mathrm{CO}}_{2}$ . Filtered ruminal fluid was mixed with the buffer solution of Menke and Steingass (1988) ( 1:2 v/v ) under a continuous flow of ${\mathrm{CO}}_{2}$ to obtain a fermentation medium. Samples of 200 mg dry matter of substrate, treated with the appropriate EFE dose, were poured into 120 mL volume serum bottles with 30 mL of the fermentation medium. Three blank samples (negative controls) were incubated without substrate to correct the produced gas from the fermentation of residual feed particles of ruminal fluid. The bottles were instantly closed with rubber stoppers and incubated in a shaking water bath at 39 ${}^{\circ }C$ for 96 h. Each treatment was performed in triplicate and repeated 3 times. Gas pressure was recorded at 2, 4, 6, 8, 12, 24, 48, 72, and 96 h by a pressure transducer (model PX4200-0100GI, Omega Engineering Inc., Laval, QC, Canada) related to a visual display transducer (Data Tracker 200, Data Track Process Instruments Ltd, Christchurch). After each measurement, the produced gas was released. The net gas was determined by subtracting the gas production from the blank bottles. The kinetics of gas production indices were adjusted by the non-linear model procedures of SAS (2011) according to the model of Ørskov and McDonald (1979) as in to Eq. (2):

2 $${\text{GP}}_{\left(t\right)}=A+B(1-e^{\left(-Ct\right)}),$$

where GP is the cumulative volume of gas produced at the time t in $\mathrm{mL}\;g^{-1}$ dry matter, t is the incubation time in h, A is the gas production from immediately soluble fraction in $\mathrm{mL}\;g^{-1}$ dry matter, B is the gas production from insoluble fraction in $\mathrm{mL}\;g^{-1}$ dry matter, A+B is the potential of gas production in $\mathrm{mL}\;g^{-1}$ dry matter, and C is the rate of gas production for the insoluble fraction in $\mathrm{mL}\;h^{-1}$ .

Metabolizable energy (ME) was estimated by Eq. (3) of Menke and Steingass (1988). The total volatile fatty acids (VFAs) were estimated according to Eq. (4) of Getachew et al. (1998):

3ME=2.2+0.136×GP+0.0057×CP4VFA=-0.00425+0.0222×GP,

where ME is the metabolizable energy value in $\mathrm{MJ}\;{\mathrm{kg}}^{-1}$ dry matter, VFAs are the ruminal total volatile fatty acids in mmol per 200 mg dry matter, CP is the crude protein in percentage of dry matter, and GP is the net gas production in mL from 200 mg after 24 h of incubation.

At 96 h of incubation, all bottles were put in ice for 5 min and the fermentation was stopped. The pH was measured immediately. The dry matter and organic matter degradability at the end of fermentation was measured according to the protocol of Elghandour et al. (2018). The partitioning factor of incubation at 24 h (PF 24) and the microbial crude protein (MCP) was determined with Eqs. (5) and (6) as described by Blümmel et al. (1997):

5 $$\text{PF 24}=\frac{\text{aDMD}}{\text{GP}},$$

where PF 24 is the partitioning factor of incubation at 24 h, GP is the net gas production in mL from 1 g dry matter after 24 h of incubation, and aDMD is the amount of dry matter digestibility in grams at the end of incubation.

6 MCP=aDMD-2.2×GP,

where MCP is microbial crude protein in $\mathrm{mg}\;g^{-1}$ dry matter, GP is the net gas production (mL) from 200 mg after 24 h of incubation, aDMD is the amount of dry matter digestibility in grams at the end of incubation, and the 2.2 $\mathrm{mg}\;{\mathrm{mL}}^{-1}$ is a stoichiometric factor that expresses the C, H, and O (measured in milligrams) required for the volatile fatty acids associated with the production of gas (measured in millilitres) (Blümmel et al., 1997).

### 2.5 Statistical analysis

Data were analysed by using a factorial model ( 4×3 ) to study the influence of four doses of EFE (control, low, medium, and high) and three substrates of olive mill waste (crude olive cake, extracted olive cake, and olive leaves). Data were analysed by using the GLM procedure of SAS (2011) following Eq. (7):

7 $$Y_{ijk}=\mu +D_{i}+S_{j}+(D\times S{)}_{ij}+E_{ijk},$$

where $Y_{ijk}$ is the observation, μ is the overall mean, $D_{i}$ is the effect of the i th dose ( i= control, low, medium, and high), $S_{j}$ is the effect of j th substrate ( j= crude olive cake, extracted olive cake, and olive leaves), ( $D\times S{)}_{ij}$ is the interaction between the dose and the substrate, and $E_{ijk}$ is the random residual error.

The orthogonal contrasts were completed to examine the linear and quadratic effects of doses for each piece of waste. Duncan's multiple range tests were performed to separate means (Duncan, 1955) and significance was confirmed at p<0.05 .

## 3 Results

### 3.1 Chemical compositions of olive mill waste

Olive mill waste is characterized by low crude protein content (89 to 102 $g\;{\mathrm{kg}}^{-1}$ dry matter), reduced sugar (9 to 57 $g\;{\mathrm{kg}}^{-1}$ dry matter), and high fibre content ( NDF>400 $g\;{\mathrm{kg}}^{-1}$ dry matter). The extracted olive cake caused the largest amount of lignocellulose waste. Olive leaves had high total polyphenols (77 $g\;{\mathrm{kg}}^{-1}$ dry matter) and condensed tannins (58 $g\;{\mathrm{kg}}^{-1}$ dry matter). Crude olive cake had a high fat content (67 $g\;{\mathrm{kg}}^{-1}$ dry matter) (Table 1).

### 3.2 Pre-incubation effects

EFE hydrolysed the fiber compound of olive leaves and olive cake. This effect was related with the rise in reduced sugars and solubility of dry matter. For the extracted olive cake, EFE failed to cause any significant difference in the chemical composition (Table 2).

### 3.3 Incubation effects

The influence of EFE on the gas production from immediately soluble and insoluble fractions, the rate of gas production for the insoluble fraction, the dry matter degradability, the organic matter degradability, the metabolizable energy value, the ruminal total volatile fatty acids, and the microbial crude protein production of olive mill waste depended on the type of the substance and the dose of EFE (Tables 3 and 4). Supplementation of EFE to crude olive cake increased gas production from immediately soluble and insoluble fractions and the rate of gas production for the insoluble fraction at all doses. The dry matter degradability, the organic matter degradability, the metabolizable energy value, the ruminal total volatile fatty acids, and the microbial crude protein production improved only at the high dose.

**Table 3:** Influence of exogenous fibrolytic enzymes on gas production kinetics' of olive mill waste.

Item	0 µL	1 µL	2 µL	4 µL	SEM	p value	Linear	Quadratic
Crude olive cake								
A	0.2 ${}^{c}$	2.2 ${}^{b}$	2.3 ${}^{b}$	3.1 ${}^{a}$	0.7	<0.0001	0.04	0.56
B	94.8 ${}^{c}$	109.7 ${}^{b}$	113.7 ${}^{b}$	130.0 ${}^{a}$	3.4	<0.0001	0.03	0.34
A+B	95.0 ${}^{c}$	111.9 ${}^{b}$	116.0 ${}^{b}$	133.1 ${}^{a}$	4.5	<0.0001	0.04	0.447
C	0.08 ${}^{b}$	0.13 ${}^{a}$	0.12 ${}^{a}$	0.14 ${}^{a}$	0.02	0.01	0.045	0.233
Extracted olive cake								
A	0.4	0.9	1.0	0.7	0.6	0.74	0.62	0.67
B	31.1	31.9	32.7	32.5	2.8	0.75	0.61	0.76
A+B	31.5	32.8	33.7	33.2	2.1	0.73	0.60	0.71
C	0.06	0.06	0.06	0.07	0.02	0.81	0.87	0.76
Olive leaves								
A	9.5 ${}^{b}$	9.4 ${}^{b}$	12.1 ${}^{a}$	8.8 ${}^{b}$	1.1	<0.0001	0.57	0.02
B	156.3 ${}^{b}$	161.0 ${}^{b}$	178.4 ${}^{a}$	145.1 ${}^{c}$	5.6	<0.0001	0.75	0.009
A+B	165.8 ${}^{b}$	170.4 ${}^{b}$	190.5 ${}^{a}$	153.9 ${}^{c}$	4.9	<0.0001	0.54	0.01
C	0.12	0.13	0.14	0.11	0.03	0.20	0.33	0.21
Item	Dose		Substrate		Dose × substrate			
p value								
A	0.01		<0.001		0.012			
B	<0.001		<0.001		0.04			
A+B	<0.001		<0.001		0.03			
C	0.03		<0.001		0.04			

${}^{\text{a, b, c}}$ Different letters in the same row indicate that the difference is significant ( p<0.05 ). SEM is the standard error of the mean. A is the gas production from immediately soluble fraction ( $\mathrm{mL}\;g^{-1}$ DM). B is the gas production from insoluble fraction ( $\mathrm{mL}\;g^{-1}$ DM). A+B is the potential of gas production ( $\mathrm{mL}\;g^{-1}$ DM). C is the rate of gas production for the insoluble fraction ( $\mathrm{mL}\;h^{-1}$ ).

**Table 4:** Effect of exogenous fibrolytic enzymes on fermentation characteristics of olive mill waste.

Item	0 µL	1 µL	2 µL	4 µL	SEM	p value	Linear	Quadratic
Crude olive cake								
pH	6.81	6.83	6.77	6.79	0.11	0.52	0.47	0.54
DMD	38.1 ${}^{b}$	41.7 ${}^{b}$	44.8 ${}^{\text{ab}}$	48.2 ${}^{a}$	3.9	0.04	0.02	0.47
DOM	40.4 ${}^{b}$	43.0 ${}^{b}$	45.2 ${}^{\text{ab}}$	49.9 ${}^{a}$	3.2	0.02	0.01	0.44
EM	4.57 ${}^{b}$	5.12 ${}^{b}$	5.32 ${}^{\text{ab}}$	5.87 ${}^{a}$	0.63	0.03	0.04	0.67
VFA	0.37 ${}^{b}$	0.46 ${}^{b}$	0.50 ${}^{\text{ab}}$	0.58 ${}^{a}$	0.10	0.03	0.04	0.67
PF 24	4.46 ${}^{b}$	3.91 ${}^{\text{ab}}$	3.93 ${}^{\text{ab}}$	3.64 ${}^{a}$	0.45	0.04	0.01	0.75
MCP	342 ${}^{c}$	367 ${}^{\text{bc}}$	393 ${}^{b}$	425 ${}^{a}$	23	0.04	0.04	0.85
Extracted olive cake								
pH	6.85	6.83	6.86	6.84	0.11	0.49	0.56	0.37
DMD	10.0	10.4	10.6	10.8	2.0	0.54	0.52	0.57
OMD	11.3	10.9	11.6	11.2	1.2	0.53	0.54	0.78
ME	2.90	2.92	2.94	2.97	0.24	0.33	0.42	0.67
VFA	0.10	0.10	0.11	0.11	0.03	0.33	0.42	0.67
PF 24	4.25	4.24	4.24	4.17	0.23	0.44	0.46	0.54
MCP	90	93	95	97	11	0.35	0.34	0.44
Olive leaves								
pH	6.79	6.79	6.75	6.80	0.07	0.62	0.56	0.49
DMD	58.3 ${}^{b}$	58.6 ${}^{b}$	66.1 ${}^{a}$	54.6 ${}^{b}$	2.5	0.02	0.34	0.04
OMD	60.4 ${}^{\text{bc}}$	62.6 ${}^{b}$	66.8 ${}^{a}$	60.0 ${}^{c}$	2.8	0.04	0.33	0.03
ME	5.76 ${}^{\text{bc}}$	5.96 ${}^{b}$	6.44 ${}^{a}$	5.40 ${}^{c}$	0.5	0.04	0.43	0.04
VFA	0.57 ${}^{\text{bc}}$	0.60 ${}^{b}$	0.68 ${}^{a}$	0.51 ${}^{c}$	0.08	0.04	0.43	0.04
PF 24	4.50	4.31	4.31	4.72	0.22	0.09	0.65	0.07
MCP	525 ${}^{b}$	527 ${}^{b}$	596 ${}^{a}$	495 ${}^{c}$	20	0.02	0.65	0.01
Item	Dose		Substrate		Dose × substrate			
p value								
pH	0.57		0.33		0.43			
DMD	0.03		<0.001		0.03			
DOM	0.03		<0.001		0.01			
ME	0.04		<0.001		0.04			
VFA	0.04		<0.001		0.04			
PF 24	0.09		0.23		0.21			
MCP	0.03		<0.001		0.01			

${}^{\text{a, b, c}}$ Different letters in the same row indicate that the difference is significant ( p<0.05 ). SEM is the standard error of the mean. pH is ruminal pH. DMD is dry matter degradability (%). OMD is organic matter degradability (%). ME is metabolizable energy value ( $\mathrm{MJ}\;{\mathrm{kg}}^{-1}$ DM) =2.2+0.136× net gas production in mL from 200 mg after 24 h of incubation+0.0057×crude protein in % dry matter. VFAs are ruminal total volatile fatty acids ( $\mathrm{mmol}\;g^{-1}$ DM) =-0.00425+0.0222×net gas production in mL from 200 mg after 24 h of incubation. PF24 is partitioning factor at 24 h of incubation (mg DMD mL ${}^{-1}$ gas). MCP is microbial crude protein production ( $\mathrm{mg}\;g^{-1}$ DM).

For olive leaves, only the medium dose increased the gas production from immediately soluble and insoluble fractions, the rate of gas production for the insoluble fraction, the dry matter degradability, the organic matter degradability, the metabolizable energy value, the ruminal total volatile fatty acids, and the microbial crude protein production. The lowest dose had no significant effect. The highest dose had a negative effect in the gas production from insoluble fraction and microbial crude protein production. For the extracted olive cake, EFE failed to cause any significant difference in nutritional value.

## 4 Discussion

### 4.1 Chemical compositions of olive mill waste

The chemical compositions of solid olive waste were comparable to the chemical composition of this waste in the review by Molina-Alcaide and Yáñez-Ruiz (2008). This waste is characterized by low crude protein ( <11 $g\;{\mathrm{kg}}^{-1}$ dry matter), a high neutral detergent fibre compound ( >400 $g\;{\mathrm{kg}}^{-1}$ dry matter), and particularly a high lignin content ( >100 $g\;{\mathrm{kg}}^{-1}$ dry matter). Crude olive cake is also characterized by high fat content, and the extracted olive cake has low fat contents. A similar result was found by Álvarez-Rodríguez et al. (2009). Olive leaves have excessive concentrations of condensed tannins. Therefore, these by-products cannot be the only components of diet but can constitute a diet supplement.

### 4.2 Pre-incubation effects

The effect of EFE depended on the substrate and the doses of EFE. For the crude olive cake, the increased dose of EFE linearly hydrolysed hemicellulose and cellulose, reduced sugars, and increased dry matter solubility. A similar result was found for fibrous feed such as wheat straw (Jabri et al., 2019) and *Dichanthium aristatum* (Díaz et al., 2015). For olive leaves, the increased dose of EFE linearly decreased the hemicellulose, increased dry matter solubility, and reduced sugars. A similar result was found on rich tannin feed like almond hulls (Abid et al., 2019b). This modification of chemical composition could modify the microorganisms in the rumen and microbial colonization. However, this additive has no effect on feeds very rich in lignin fraction like extracted olive cake. Indeed, this component may prevent the contact of EFE with other commands of substrate (Hatfield et al., 1999). The second extraction of crude olive cake particularly increased the lignin fraction by 102 $g\;{\mathrm{kg}}^{-1}$ dry matter. The excessive concentration of this compound can block the effect of enzymes.

### 4.3 Incubation effects

The response to EFE depended on the substrate and the doses of EFE. This additive modified the nutritional value of crude olive cake and olive leaves. However, it did not affect nutritional value of extracted olive cake. The absence of an effect on the chemical composition in the pre-incubation period explained the lack of effect on the nutritional value of this substrate. For the crude olive cake, the increased dose of EFE linearly increased the gas production from immediately soluble and insoluble fractions, the gas production from insoluble fraction, the rate of gas production for the insoluble fraction, the dry matter degradability, the organic matter degradability, the metabolizable energy value, the ruminal total volatile fatty acids, and the microbial crude protein production. Similar results were found in fibrous tropical foraging material (Sakita et al., 2020). These improvements in fermentation and degradability cannot be explained exclusively by the small hydrolysed quantity of cellulose and hemicellulose ( <4 % of dry matter). It was highly probable that these improvements were due to the synergy between EFE and the ruminal flora. The increase in microbial production of crude protein following the use of this additive is mainly due to better accessibility of nutrients for rumen microorganisms (Getachew et al., 2004). It was very likely that this improvement was due to the increase in ruminal bacteria. In fact, Newbold et al. (1992) have shown that EFE produced from *Aspergillus oryzae* (Amaferm; BioZyme Enterprises Inc., St Joseph, MO, USA) can stimulate total bacterial numbers by 34 % and cellulolytic bacterial numbers by 90 % in rumen fluid (Newbold et al., 1992). Similarly, Salem et al. (2015) showed an increase of 43 % in microbial crude protein production of corn silage and concentrate supplemented with the same mixture used in this study. Moreover, this enzymatic additive increased the production of volatile fatty acids. A similar effect was demonstrated for straws treated with xylanase (Sujani et al., 2015). The increased rate of fermentation of the insoluble fraction following the use of this additive may be accelerated by the feed transit in the rumen and consequently increase the energy intake of low-energy feed. For olive leaves, the increased dose of EFE modified the gas production from immediately soluble and insoluble fractions, the dry matter degradability, the organic matter degradability, the metabolizable energy value, the total volatile fatty acids, and the microbial crude protein production. Only the medium dose increased the nutritional value of this waste. The high dose significantly decreased the gas production from the insoluble fraction by 7 % and microbial crude protein production by 6 % compared with the control. The negative effect of the highest dose had also been proven in vivo (Kung et al., 2000) and in vitro (Abid et al., 2019a, b). This inhibiting effect may be due to the release of anti-nutritional elements of the olive leaves, like the phenolic compounds, in the ruminal environment. The latter at high concentrations may inhibit the proliferation of rumen flora and partially reduce the activity of enzymes (Treacher and Hunt, 1996; Molina-Alcaide and Yáñez-Ruiz, 2008). The high quantity of the xylan and xylose released in the pre-incubation period may be causing furfural (Mosier et al., 2005) and inhibited proliferation of rumen flora (Castro et al., 1994). The excessive dose of EFE may block the attachment sites of ruminal microorganisms and the action sites of the endogenous zone (Nsereko et al., 2000; McAllister et al., 2001; Eun and Beauchemin, 2008).

## 5 Conclusions

The EFE hydrolysed cell-wall components, reduced sugars, and increased the dry matter solubility of crude olive cake and olive leaves. Upon in vitro incubation, it increased the fermentation, the degradability, the metabolizable energy value, the volatile fatty acids, and the microbial crude protein production. Further research work in these areas is needed to verify the effectiveness of these enzymatic complexes with other fibrous waste.

## Data Availability

The software code as well as the original data of the paper is available from the corresponding author upon request.
